# Processing of Neutrophil α-Defensins Does Not Rely on Serine Proteases *In Vivo*

**DOI:** 10.1371/journal.pone.0125483

**Published:** 2015-05-06

**Authors:** Andreas Glenthøj, Katrin Nickles, Jack Cowland, Niels Borregaard

**Affiliations:** 1 The Granulocyte Research Laboratory, Department of Hematology, Rigshospitalet, University of Copenhagen, Denmark; 2 Poliklinik für Parodontologie, Zentrum der Zahn-, Mund- und Kieferheilkunde (Carolinum), Johann Wolfgang Goethe-Universität, Frankfurt, Germany; University of Tübingen, GERMANY

## Abstract

The α-defensins, human neutrophil peptides (HNPs) are the predominant antimicrobial peptides of neutrophil granules. They are synthesized in promyelocytes and myelocytes as proHNPs, but only processed in promyelocytes and stored as mature HNPs in azurophil granules. Despite decades of search, the mechanisms underlying the posttranslational processing of neutrophil defensins remain unidentified. Thus, neither the enzyme that processes proHNPs nor the localization of processing has been identified. It has been hypothesized that proHNPs are processed by the serine proteases highly expressed in promyelocytes: Neutrophil elastase (NE), cathepsin G (CG), and proteinase 3 (PR3), all of which are able to process recombinant proHNP into HNP *in vitro*. We investigated whether serine proteases are in fact responsible for processing of proHNP in human bone marrow cells and in human and murine myeloid cell lines. Subcellular fractionation of the human promyelocytic cell line PLB-985 demonstrated proHNP processing to commence in fractions containing endoplasmic reticulum. Processing of ^35^S-proHNP was insensitive to serine protease inhibitors. Simultaneous knockdown of NE, CG, and PR3 did not decrease proHNP processing in primary human bone marrow cells. Furthermore, introduction of NE, CG, and PR3 into murine promyelocytic cells did not enhance the proHNP processing capability. Finally, two patients suffering from Papillon–Lefèvre syndrome, who lack active neutrophil serine proteases, demonstrated normal levels of fully processed HNP in peripheral neutrophils. Contradicting earlier assumptions, our study found serine proteases dispensable for processing of proHNPs *in vivo*. This calls for study of other protease classes in the search for the proHNP processing protease(s).

## Introduction

Neutrophils are of paramount importance for the ability to fight invading microorganisms. Their antimicrobial activity relies partly on a range of antimicrobial peptides localized in granules. Neutrophil α-defensins, also known as human neutrophil peptides (HNPs), are small antimicrobial peptides with antibacterial, antiviral, and antifungal activities. In humans, HNPs are the most abundant of all neutrophil granule proteins. They constitute 5–7% of the total neutrophil protein and 30–50% of azurophil granule protein[1].

**Fig 1:**
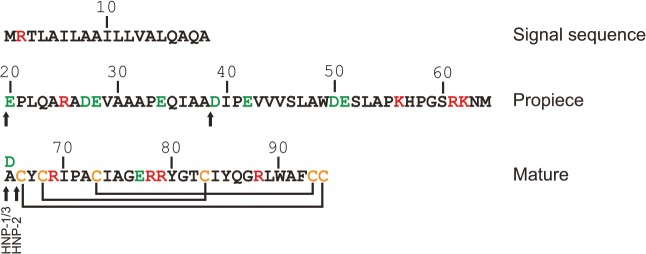
Structure of preproHNP-1-3. Arrows indicate major sites of proteolytic cleavage. Positively and negatively charged amino acids are indicated in red and green, respectively. Lines indicate the disulphide linkage of cysteines (C; orange). HNP-3 is identical to HNP-1 except for having substituted alanine (A) at position 65 for aspartic acids (D).

Neutrophil α-defensins are generated as 75 amino acids (aa) pro-peptides with an N-terminal prosegment having a negative charge that neutralizes the highly positively charged C-terminus (Fig 1). Processing of α-defensins occurs mainly in late promyelocytes, where the 75 aa proHNPs are cleaved to a 56 aa intermediate form and onward to 29–30 aa mature peptides designated HNPs[2,3]. Cationic 29–30 aa HNPs associate with the negatively charged proteoglycan serglycin and translocate to azurophil granules[4]. In later stages of granulocytic differentiation in which HNP expression peaks, proHNPs are not cleaved and most proHNPs are secreted into the bone marrow (BM) plasma although a minor fraction is retained in specific granules[5,6].

The intestinal α-defensins, which are generated in Paneth cells in the crypts of Lieberkühn, are processed by matrix metalloprotease-7 (matrilysin) in mice and by the serine protease trypsin in humans[7,8]. The enzyme responsible for processing of neutrophil α-defensins *in vivo* is currently unknown, but *in vitro* studies have shown recombinant proHNP to be fully processed by neutrophil elastase (NE) and proteinase 3 (PR3), and partially processed by cathepsin G (CG)[9,10]. *In vivo* models are complicated by the lack of neutrophil defensins in wild type mice[11,12]. In the transgenic HNP-1 mouse, NE is dispensable for processing of proHNP[4]. NE, PR3, and CG are serine proteases highly expressed during the promyelocytic stage of neutrophil differentiation, but their expression ceases abruptly[13] along with proHNP processing when the cells mature to myelocytes. These serine proteases are therefore prime candidates as the proHNP processing enzymes.

Serine proteases such as NE, PR3, and CG share a high degree of homology. As zymogens, they contain a prodipeptide between the signal peptide and the mature active enzyme[14]. Removal of this prodipeptide, which is executed by cathepsin C, also known as dipeptidyl peptidase I, is required for activation of the proteases[15–17]. Patients with the Papillon–Lefèvre syndrome (PLS) have inactivating cathepsin C mutations and hence their neutrophils lack serine protease activity[16,17]. Clinically, the patients suffer from severe periodontitis and palmoplantar keratoderma, but are not prone for systemic infections[17].

We hypothesized that proHNPs are processed before translocation to granules by a protease only expressed in promyelocytes. We created a novel assay for proHNP processing activity and performed subcellular fractionation of human promyelocytic cells to identify fractions in which processing occurs. Furthermore, we examined whether NE, PR3, or CG are responsible for proHNP processing *in vivo*. For this purpose, we utilized promyelocytic cell lines as well as primary human BM cells. Finally, we examined blood samples from two patients with PLS as an *in vivo* model for lack of serine proteases.

## Materials and Methods

### Ethical statement

The Ethics Committee of the Capital Region of Denmark specifically approved the study (H-1-2011-165). BM aspirates and peripheral blood (PB) plasma were obtained from healthy donors giving informed written consent according to the permission and guidelines from the Ethics Committee of the Capital Region of Denmark (H-1-2011-165). PB was obtained from PLS patients after giving informed written consent according to the permission and guidelines from the Institutional Review Board for Human Studies of the Medical Faculty of the Johann Wolfgang Goethe-University Frankfurt/Main (#31/05).

### Isolation of neutrophils

Neutrophils were isolated from peripheral blood by density centrifugation and subsequent hypotonic lysis of contaminating erythrocytes as previously described[18].

### Subcellular fractionation

**Fig 2:**
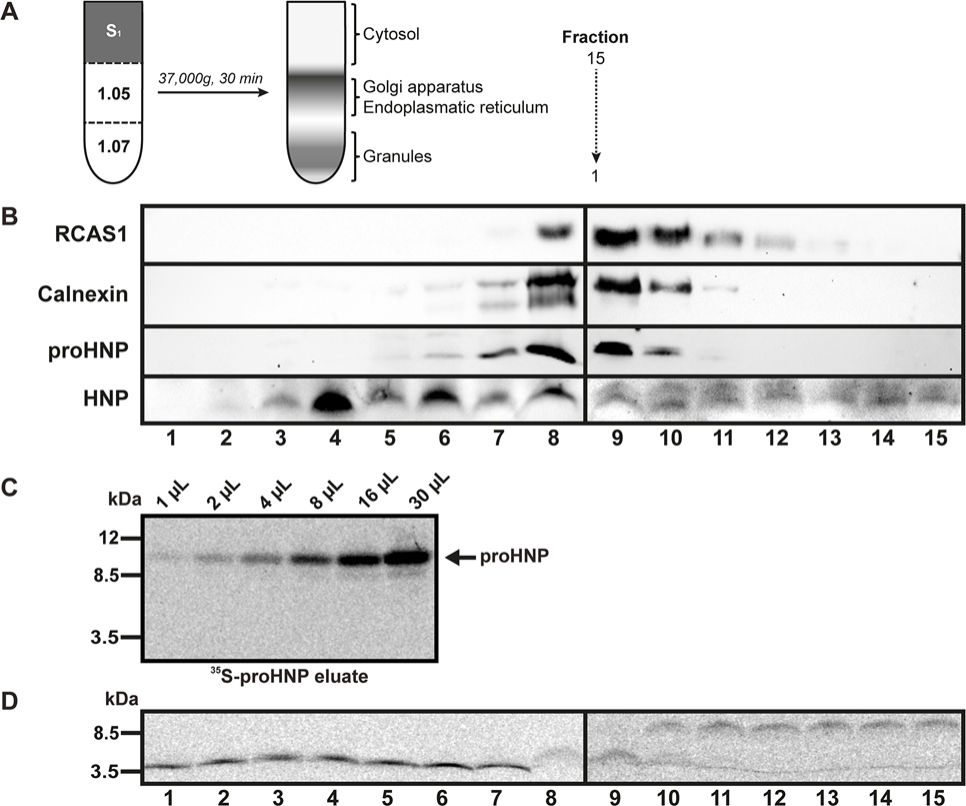
Subcellular localization of proHNP processing. (A) PLB-985 cells were pelleted and disrupted by nitrogen cavitation. After low speed centrifugation, the cavitate was divided in a postnuclear pellet (P_1_) consisting of nuclei and unbroken cells and a post-nuclear supernatant (S_1_) containing cytosol, organelles (including granules), and cell membranes. S_1_ was underlaid with a two-layer 1.05/1.07 PBS/Percoll density gradient and centrifuged at 37.000*g* for 30 minutes. Fractions were collected from the bottom of the gradient. Percoll was removed from fractions by ultracentrifugation. (B) Fractions were subjected to Western blotting for HNP, proHNPs, the endoplasmic reticulum (ER) marker calnexin, and the Golgi marker RCAS1. (C) PLB-985 cells were pulsed overnight in medium containing 100 μCi/mL ^35^S-methionine/cysteine. Cells were pelleted and the supernatant used for isolation of ^35^S-labelled proHNP by affinity chromatography with an antibody specific for proHNP. Radioactive fractions were pooled, dialyzed against PBS, and tested for proHNP by 16% SDS-Tricine-PAGE and fluorography. (D) ^35^S-proHNP was incubated with subcellular fractions of PLB-985 for 15 hours at 37°C. Processing was tested by 16% SDS-Tricine-PAGE and fluorography.

PLB-985 cells were pelleted and disrupted by nitrogen cavitation. After centrifugation at 400*g* for 15 minutes, the cavitate was divided in a nuclear pellet (P_1_) consisting of nuclei and unbroken cells and a post-nuclear supernatant (S_1_) containing cytosol, organelles (including granules), and cell membranes. To obtain subcellular fractions, S_1_ was underlaid with a two-layer 1.05/1.07 Percoll density gradient and centrifuged at 37.000*g* for 30 minutes (Fig 2A). Fractions were collected from the bottom of the gradient. Percoll was removed from fractions by ultracentrifugation at 200.000*g* for 45 minutes on Optima L-100 XP equipped with a 50.4TI rotor head (Beckman Coulter). To obtain a pool of promyelocytic proteases for proHNP processing assay, S_1_ was centrifuged at 20.000*g* for 30 minutes and the pellet (P_2_) was solubilised in PBS/1% Triton X-100.

### Cell culture

PLB-985 cells[19] (a kind gift from Dr. Peter Newberger, University of Massachusetts Medical School) were cultured in RPMI-1640 medium with Glutamax, 20% fetal calf serum (FCS), 100 U/mL penicillin, and 100 μg/mL streptomycin (all from Invitrogen) in a humidified incubator with 5% CO_2_ at 37°C. MPRO cells (CRL-11422, ATCC) were cultured in AIM-V with 20% horse serum (Invitrogen), 5% conditional media from HM5 cells, Glutamax, 100 U/mL penicillin, and 100 μg/mL streptomycin (all from Invitrogen) in a humidified incubator with 5% CO_2_ at 37°C. HM5 cells[20] were generously provided by Dr. Nancy Berliner, Harvard Medical School and cultured in RPMI 1640 with Glutamax, 10% FCS, 100 U/mL penicillin, and 100 μg/mL streptomycin.

### Transfection

The coding sequences of *DEFA1*, *ELANE*, *CTSG*, and *PRTN3* were cloned into the mammalian expression vector pEF6/*myc*-His A (*DEFA1* only; Invitrogen) or pEF1/V5-His A (Invitrogen) as previously described[4]. The following primers were used for amplification of *CTSG*: 5’-ATGGTACCGCCACCATGCAGCCACTCCTGCTTCT-3’ and 5’-ATTCTAGATCACAGGGGGGTCTCCATC-3’. 5x10^6^ MPRO cells were resuspended in 100 μL Ingeneo Electroporation Solution (Mirus Bio), mixed with 2 μg plasmid DNA, and electroporated in an Amaxa Nucleofector (Lonza) using Program T-20. Cells were transferred to 5 mL of cell-culture medium and allowed to recover in a humidified incubator with 5% CO2 at 37°C. After 2 days, 50 mL of culture medium and selection antibiotic were added to a final concentration of 15 μg/mL blasticidin (Invitrogen) or 1 mg/mL G418 (Invitrogen). Cells were plated in wells containing 1 mL each and grown under selection pressure for 3 weeks. Wells positive for living cells after 3 weeks were grown in medium supplemented with selection antibiotics.

SiRNA mediated knockdown was performed on human primary bone marrow cells as previously described[4] using siRNA against *ELANE* (s4601), *CTSG* (s3745), and *PRTN3* (s11286; all from Life Technologies).

### Isolation of BM cells

Murine BM cells and granulocytic precursors from human BM were obtained as previously described[4].

### Real-time quantitative PCR

RNA isolation and cDNA synthesis were performed as previously described[21]. cDNA was subjected to quantitative real-time polymerase chain reaction (PCR) analysis using TaqMan gene expression assays (Applied Biosystems) on a 7500 Real-Time PCR system, according to the manufacturer’s instructions. Assays included: *DEFA1* (Hs00234383_m1), *ELANE* (Hs00236952_m1), *CTSG* (Hs00175195_m1), *PRTN3* (Hs00157572_m1). Expression levels were normalized to the constitutively expressed housekeeping mouse gene *Gapdh* (4352339E) or human gene *GAPDH* (4326317E). Tests were performed in triplets. Standard deviations were calculated by Stratagene MxPro 4.1.

### Pulse-chase biosynthesis

Pulse-chase biosynthesis was performed as previously described[4].

### ^35^S-ProHNP processing assay

1x10^8^ PLB-985 cells were pelleted and resuspended at 2x10^7^ cells/mL in DMEM without L-methionine/L-cysteine (Invitrogen) containing 100 U/mL penicillin, 100 μg/mL streptomycin, and 10% dialyzed FCS, and incubated for 40 minutes at 37°C. The cells were pelleted by centrifugation, resuspended at 5x10^6^ cells/mL in DMEM as above to which ^35^S-methionine/cysteine (NEG772002MC; Perkin Elmer) had been added to a final concentration of 100 μCi/mL, and pulsed for overnight in a humidified incubator with 5% CO_2_ at 37°C. Cells were pelleted and the supernatant used for isolation of ^35^S-proHNP by affinity chromatography as previously described[22]. Small aliquots of eluate were tested for radioactivity on a Fuji BAS2500 PhosphorImager (Fuji Film). Radioactive fractions were pooled and dialyzed against PBS.

Processing assay was performed by incubating ^35^S-proHNP with analyte in Dulbecco's Phosphate-Buffered Saline (DPBS; Life Technologies) at 37°C. Assay was stopped by addition of Laemmli buffer[23]. This was subjected 16% SDS-Tricine-PAGE, stained with Coomassie, soaked in Amplify (NAMP100V; GE Healthcare), placed in a Fuji BAS cassette (Fuji Film), and developed by a Fuji BAS2500 PhosphorImager.

11 mM diisopropyl fluorophosphate (DFP; Merck Millipore), 1 mM phenylmethanesulfonylfluoride (PMSF; Sigma), or 1μM elastase inhibitor IV (Merck Millipore) were added to inhibit serine proteases.

### Enzyme activity assays

Subcellular fractions were lysed in PBS/1% Triton X-100 and PLS neutrophils lysed in 100 mM phosphate buffer (pH 6.2)/0.2% Triton X-100. Assays were performed as previously described[24].

### Antibodies

The following antibodies were used: rabbit anti-calnexin (ab10286, Abcam), and rabbit anti-RCAS1 (#12290, Cell Signaling Technology), rabbit anti-proHNP[5], rabbit anti-HNP[25], rabbit anti-NE[4], rabbit anti-PR3[26], rabbit anti-CG (A588, Dako), goat anti-β-actin (sc-1616, Santa Cruz Biotechnology), and rabbit control IgG (X0903; Dako). The 45 aa propiece of proHNP-1–3 (EPLQARADEVAAAPEQIAADIPEVVVSLAWDESLAPKHPGSRKNM) was synthesized by Schaefer-N, Copenhagen, Denmark. Rabbit antibodies against this synthetic peptide were generated by Dako through their contract immunization program. The antibodies are specific for proHNPs[10].

### Western blotting

SDS-Tricine-PAGE and immunoblotting were performed as previously described[4].

## Results

### ProHNP processing is initiated in pre-granular fractions

We performed subcellular fractionation on promyelocytic PLB-985 cells in order to identify fractions containing proHNPs and mature HNPs (Fig 2A). ProHNPs showed a distribution similar to the ER marker calnexin (fractions 6–10), whereas processed HNPs were found in fractions 3–8 with peak in fraction 4 (Fig 2B). This indicated that proHNP-processing commences in ER fractions.

To test if proHNP processing enzymes were present in these fractions, we developed a novel proHNP processing assay. ^35^S-labelled proHNP was obtained by affinity chromatography of medium from promyelocytic PLB-985 cells incubated overnight in medium containing ^35^S-cysteine/methionine (Fig 2C). ^35^S-proHNP was incubated at 37°C with subcellular fractions from PLB-985 cells for 15 hours, subjected to SDS-Tricine-PAGE, and visualized by fluorography (Fig 2D). Fractions 1–7 were able to completely process proHNP after 15 hours incubation, while fractions 8–9 only showed intermediate processing. This is in accordance with proHNP processing starting in ER-fractions.

### ProHNPs can in vitro be processed by non-serine promyelocytic proteases

**Fig 3:**
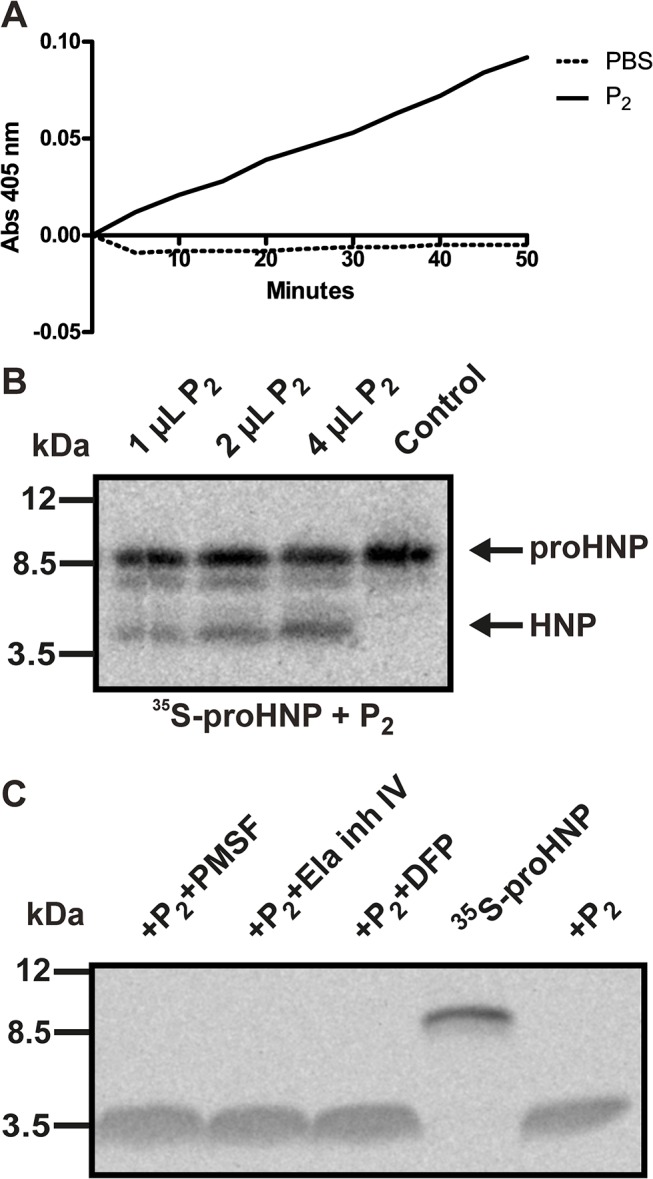
^35^S-proHNP processing assay using promyelocytic PLB-985 proteases. (A) PLB-985 cells was subjected to nitrogen cavitation followed by pelleting of the postnuclear supernatant containing cytosol, organelles (including granules), and cell membranes. This pellet (P_2_) was permeabilized in PBS/1% Triton X-100. Preservation of serine protease activity was verified by measuring elastase/proteinase 3 activity by spectrophotometry following degradation rate of methoxysuccinyl-Ala-Ala-Pro-Val-P-nitroanilide. (B) ^35^S-proHNP was incubated with P_2_ from PLB-985 cells, incubated at 37°C for 3 hours, and processing tested by SDS-Tricine-PAGE followed by fluorography. (C) P_2_ was mixed with the serine proteinase inhibitors DFP, PMSF, or elastase inhibitor IV, mixed with ^35^S-proHNP, and incubated for 6 hours. Processing was analyzed by SDS-Tricine-PAGE followed by fluorography. Complete inhibition of serine proteases by DFP was verified by protease inhibition assays (data not shown).

We next isolated membrane enclosed organelles from the promyelocytic cell line PLB-985, assuming that proHNP processing proteases would be present in these. PLB cells were disrupted by nitrogen cavitation and the postnuclear supernatant which contains cytosol, organelles (including granules), and cell membranes was centrifuged to sediment the membrane bound organelles (P_2_). Serine protease activity of P_2_ was confirmed by a NE/PR3 assay (Fig 3A).

P_2_ from PLB-985 cells were fully capable of processing ^35^S-proHNP after 3 hours of incubation at 37°C (Fig 3B). In accordance with *in vivo* conditions[27], an intermediate was also observed during proHNP processing (Fig 3B).

Next, we tested whether processing of proHNP by proteases present in promyelocytes could be inhibited by the broad serine proteinase inhibitors DFP and PMSF as well as the selective elastase inhibitor IV (Fig 3C). As seen, neither serine protease inhibitor prevented processing of proHNP. Complete serine protease inhibition by DFP was verified by protease activity assay (data not shown).

### Serine proteases knockdown in primary human BM cells does affect proHNP processing

**Fig 4:**
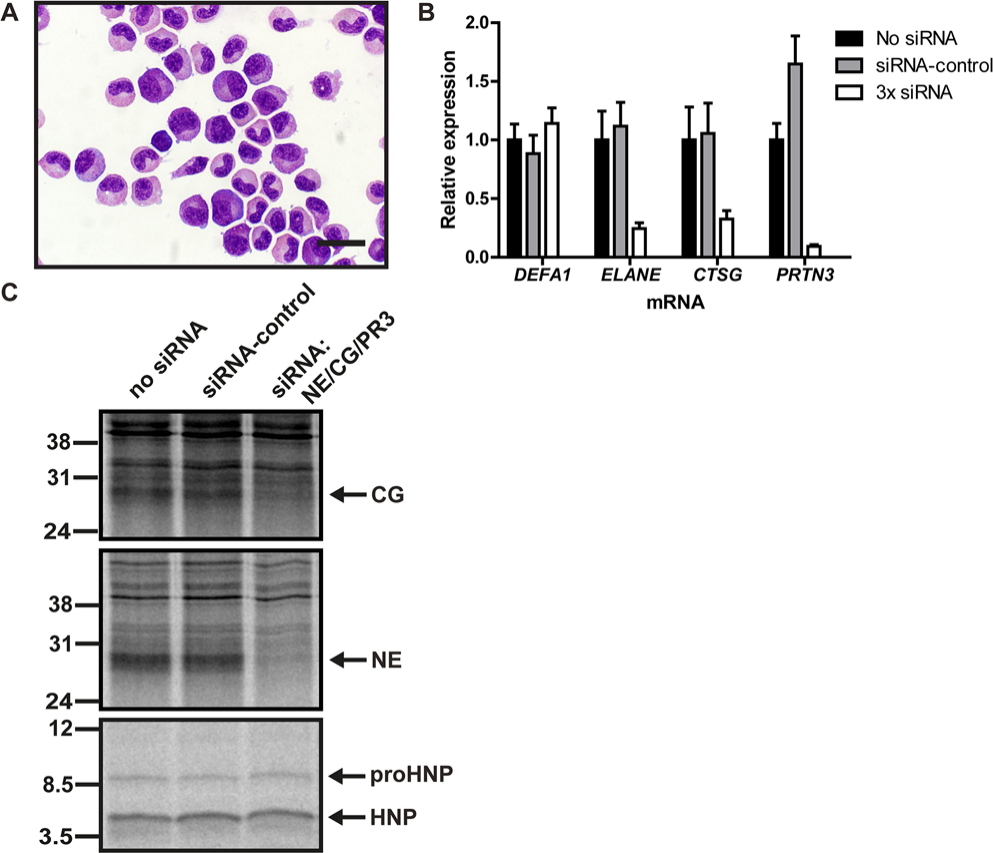
Biosynthesis of proHNP and HNP in human bone marrow. (A) Human bone marrow cells were sedimented with dextran. Supernatant was laid on Lymphoprep and centrifuged at 400*g* for 30 minutes. Interphase cells were depleted of nongranulocytic cells by immunomagnetic sorting, spun onto slides and May-Grünwald Giemsa stained. Bar represents 20 μm. (B) Purified granulocytic precursors were electroporated with 3x siRNA (against neutrophil elastase (NE; *ELANE*), cathepsin G (CG; *CTSG*), and proteinase 3 (PR3; *PRTN3*)), control siRNA, or without siRNA and incubated for 24h in a humidified incubator with 5% CO_2_ at 37°C. Comparative quantification mRNA for *DEFA1*, *ELANE*, *CTSG*, and *PRTN3* was performed by real-time PCR. Figure depicts expression levels relative to cells electroporated without siRNA. Bars represent means and lines represent standard deviation. (C) Transfected granulocyte precursors were pulsed with ^35^S-methionine/cysteine for 2 hours and chased overnight. Cell lysates and medium were immunoprecipitated with antibodies in the following order: anti-proHNP, anti-HNP, anti-CG, anti-NE, and anti-PR3. CG (top) and NE (mid) immunoprecipitates were analyzed by 12% SDS-PAGE and fluorography. Immunoprecipitation with anti-PR3 did not yield a specific PR3 band (data not shown). ProHNP and HNP immunoprecipitates were pooled and analyzed by 16% SDS-Tricine-PAGE and fluorography (bottom).

We isolated granulocytic precursors from human BM (Fig 4A) and transiently transfected these with siRNAs against NE (*ELANE*), CG (*CTSG*), and PR3 (*PRTN3*) and achieved a simultaneous knockdown of 76%, 67%, and 91% respectively without affecting HNP-1 expression (Fig 4B). Twenty-four hours after transfection, cells were subjected to pulse-chase biosynthesis (Fig 4C). NE and CG knockdown was verified on protein level. ProHNP processing was unaffected of the significant reduction of serine proteases.

### Transfection of serine proteases into promyelocytic cells does not enhance proHNP processing

**Fig 5:**
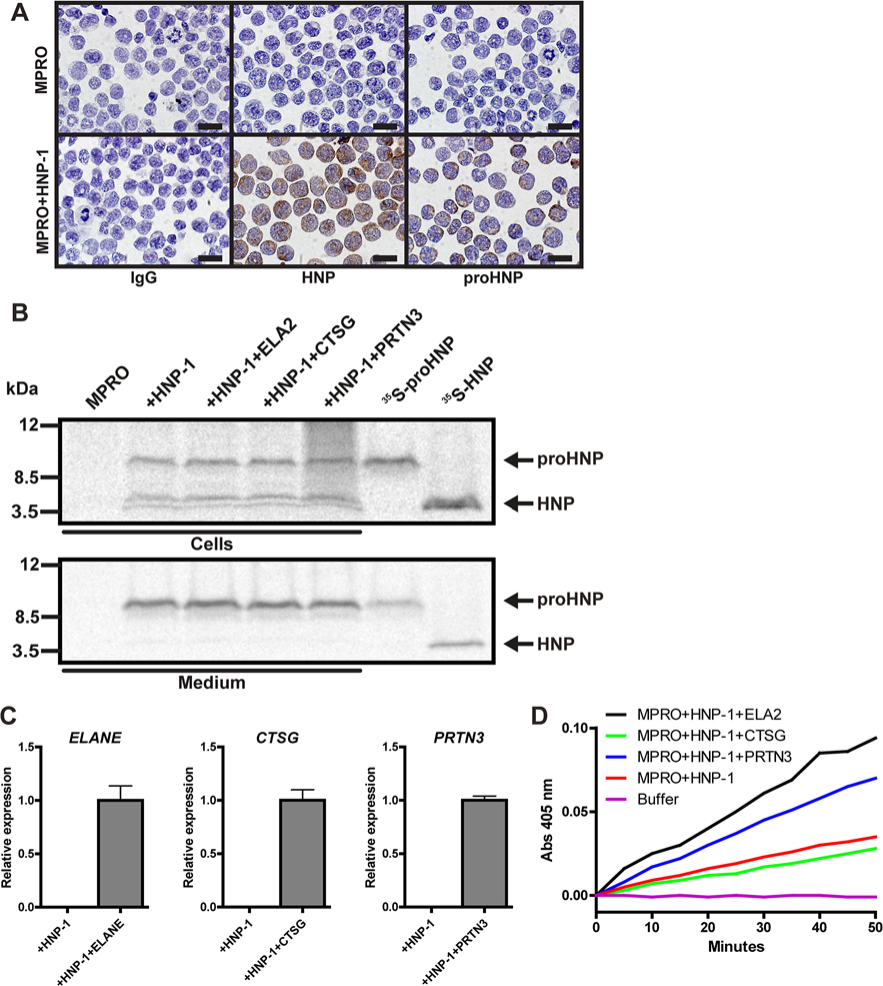
Processing of proHNP-1 by MPRO cells transfected with human serine proteases. (A) MPRO cells and MPRO cells transfected with HNP-1 were spun onto slides, fixed, permeabilized, and immunocytochemically stained for IgG (top), HNPs (middle), and proHNPs (bottom). Bars represent 20 μm. (B) MPRO-HNP-1 cells were further transfected with the human neutrophil serine proteases neutrophil elastase (*ELANE*), cathepsin G (*CTSG*), and proteinase 3 (*PRTN3*). Cells were pulsed with ^35^S-methionine/cysteine for 1 hour and chased overnight. Cell lysates and medium were immunoprecipitated with antibodies in the following order: anti-proHNP, and anti-HNP. Immunoprecipitates were pooled and analyzed by 16% SDS-Tricine-PAGE and fluorography using ^35^S-proHNP and ^35^S-HNP from the proHNP processing assay as controls. (C) Comparative quantification mRNA for *ELANE*, *CTSG*, and *PRTN3* was performed by real-time PCR. Figure depicts expression levels relative to cells electroporated human serine proteases. Bars represent means and lines represent standard deviation. (D) Activity of transfected human neutrophil elastase and proteinase 3 was asserted by lysis of transfected MPRO cells followed by spectrophotometry following degradation rate of methoxysuccinyl-Ala-Ala-Pro-Val-P-nitroanilide.

The mouse promyelocytic cell line MPRO was transfected with a plasmid construct containing an expression cassette into which the coding sequence of the HNP-1 gene had been inserted. The transfected cells clearly showed reactivity with antibodies against HNPs purified from human neutrophil azurophil granules and against recombinant prosegment of HNP-1 (Fig 5A). Pulse-chase biosynthesis demonstrated that MPRO cells process the proHNP-1 into a small peptide of similar size as HNPs from human neutrophils (Fig 5B). MPRO cells already transfected with HNP-1 were transfected with a plasmid expressing either human NE, CG, or PR3 to test if high expression of these proteases would increase the rather moderate processing of proHNP-1 in MPRO cells. Transfection was verified by real-time PCR (Fig 5C). Cells transfected with NE or PR3 exhibited increase activity on protease assay of NE/PR3 (Fig 5D) demonstrating intact activation of the serine proteases. Double transfection did not result in increased proHNP-1 processing as judged by pulse-chase biosynthesis studies (Fig 5B).

### Serine protease activity is not necessary for proHNP processing in vivo

**Fig 6:**
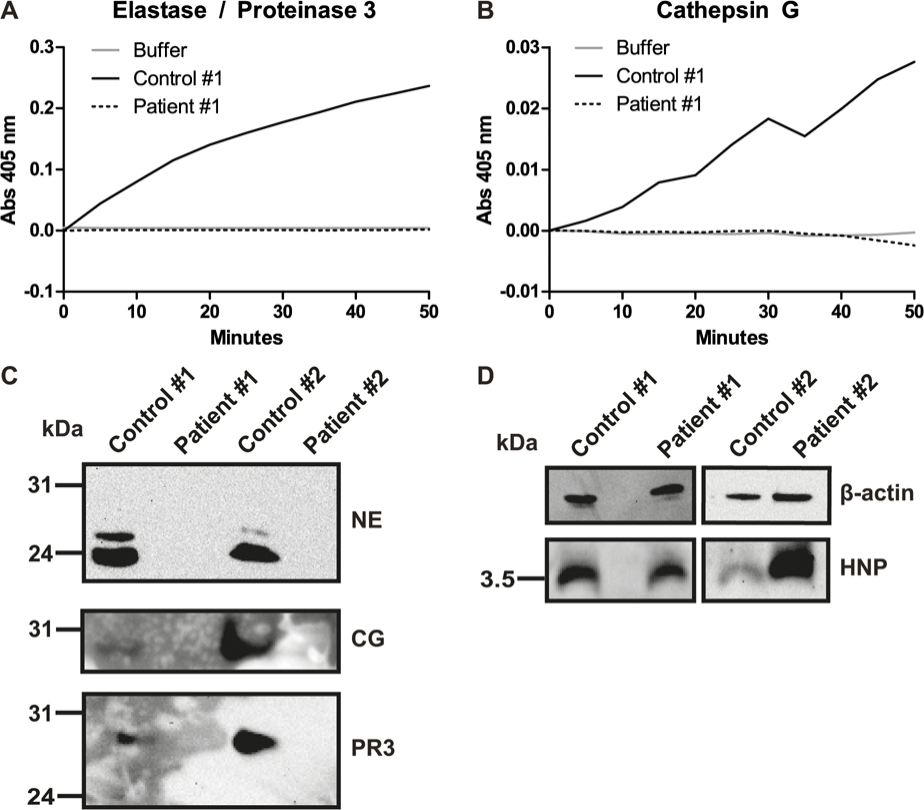
Serine protease activity is not required for HNP processing *in vivo*. Peripheral blood neutrophils from two patients suffering from Papillon–Lefèvre syndrome (PLS). (A) Neutrophil elastase (NE) and proteinase 3 (PR3) activity was measured as absorbance (Abs) following degradation of methoxysuc-AAPV-p-nitroanilide. Assay from patient 1 is shown and is representative of both patients. (B) Cathepsin G (CG) activity was measured as absorbance following degradation of N-suc-AAPF-p-nitroanilide. Assay from patient 1 is shown and is representative of both patients. (C) Western blotting of NE, CG, and PR3 in two PLS patients and controls. (D) Western blotting of HNP and β-actin in two PLS patients and controls.

Peripheral blood was obtained from two patients with genetically verified PLS (Table 1). Both patients have previously been described and shown to have no activity of cathepsin C, NE, CG, or PR3 in neutrophils[28,29]. In accordance with previous results[29], enzyme activity assays on neutrophils from both patients showed no activity of NE, PR3, or CG (Fig 6A and 6B). Furthermore, Western blotting showed no NE, CG, or PR3 in PLS neutrophils (Fig 6C). However, patient neutrophils were not deficient in fully processed HNPs demonstrating that serine proteases are dispensable for processing of proHNPs *in vivo* (Fig 6D). This is in line with the recent finding obtained by subcellular fractionation of a patient with PLS[30].

**Table 1:** Papillon-Lefévre patients.

Patient	Nucleotide	Exon	Effect
1	947 T>G, 1268 G>C	7	L316R, W423S
2	854 C>T	6	P285L

Mutations of the Papillon-Lefévre patients. Nucleotides are numbered according to the coding DNA sequence (CDS).

## Discussion

Posttranslational processing of neutrophil defensins is complex and the processing protease(s) has escaped identification for almost three decades. It has been assumed that proHNPs are cleaved by one or more protease(s) synthesized only in promyelocytes[31], which would explain the lack of processing at later stages of granulopoiesis[4]. Obvious candidates that fit these requirements are the neutrophil serine proteases NE, PR3, and CG, which are synthesized in high amounts in promyelocytes, and which are all capable of processing proHNPs in a test tube[9,10]. Our aim was to test, whether this is also the case *in vivo*.

We found that proHNP processing in promyelocytic cells commences in fractions containing ER and continues throughout Golgi containing fractions, whereas granule fractions totally lacked proHNPs (Fig 2). Our findings extend those of an early study in which 75 aa proHNPs was only found in the microsomal fraction of cells from patients with chronic myeloid leukemia, whereas an intermediate and mature HNP was found in both the microsomal and granule fraction[2]. This indicates that at least the initial cleavage of proHNP is carried out by one or more proteases residing in ER and Golgi during the promyelocytic stage of neutrophil differentiation. Pre-granular processing also correlates well with a recent report demonstrating that mature HNPs, but not proHNPs, are retained intracellularly by electrostatic interaction with the anionic proteoglycan serglycin[4], which is located in the Golgi apparatus[32].

We developed a novel ^35^S-proHNP processing assay, which is highly sensitive and not influenced by the presence of endogenous defensins. Studies with DFP, a potent and irreversible inhibitor of serine proteases, demonstrated that non-serine promyelocytic protease were also capable of processing proHNPs *in vitro* (Fig 3). In mice, intestinal pro-α-defensins can be activated by a range of proteases including microbial proteases[33]. It is possible that several proteases expressed by promyelocytes can process proHNPs. This would hinder identification of the processing proteases by single knockdown or knockout models. Our ^35^S-proHNP processing assay opens the possibility of high-throughput screening of processing capability of promyelocytic proteases.

Simultaneous knockdown of serine proteases in primary granulocytic precursors from human bone marrow had no effect on the posttranslational processing of proHNPs (Fig 4). As proHNPs are very highly expressed, one would expect that a knockdown in the range 67–91% of the processing protease(s) would at least somewhat diminish the processing capability of the cells. Similarly, introduction of human NE, CG, or PR3 into MPRO cells transfected with proHNP-1 did not improve their limited capacity to process proHNP-1 (Fig 5). Finally, we obtained peripheral blood from patients with PLS that lack functional cathepsin C, and therefore cannot activate neutrophil serine proteases[17]. In accordance with earlier findings[17], the PLS patients not only lacked serine proteases activity, the proteases themselves were also absent. This indicates that cleavage of the amino-terminal propiece by cathepsin C may be necessary for retention of neutrophil serine proteases intracellularly[34]. Notably, these patients had no deficit in mature HNP in neutrophils demonstrating that serine proteases are not essential for processing of neutrophil defensins *in vivo*. As shown in a recent report[35], this also pertains to the newly described neutrophil serine protease NSP4. However, our study does not exclude the possibility that neutrophil serine proteases process proHNPs *in vivo*, but our results demonstrate that the serine proteases are not necessary for processing *in vivo*.

In mice, deficiencies in NE render the mice prone to common bacterial infections such as Klebsiella pneumonia, Staphylococcus aureus, and Escherichia coli[36,37]. Furthermore combined NE/CG knockout mice are also deficient in combatting mycobacteria and systemic fungal infections[38,39]. In contrast, the immunodeficiency of PLS patients is mostly limited to the oral cavity, despite lack of all neutrophil serine proteases[40]. *In vitro*, neutrophils from PLS patients are not uniformly deficient in killing common bacteria[40] suggesting that human neutrophils possess alternative killing pathways. HNPs have a variety of antimicrobial functions *in vitro*, including activities against Staphylococcus aureus, Pseudomonas aeruginosa, and Escherichia coli[41]. Proving this *in vivo* has been difficult as lack of HNPs has not been described in humans[42] and mice do not express functional myeloid defensins[43]. As demonstrated by us, PLS patients have intact capacity to synthesize and store HNPs and it is possible that this “rescues” the phenotype due to lack of serine proteases with respect to infections and that the presence of HNPs in humans may explain some of the striking discordance of immunodeficiency in humans and mice deficient in neutrophil serine proteases. Neutrophil serine proteases are implicated in the destruction of tissue in a wide range of diseases such as acute respiratory distress syndrome (ARDS), chronic obstructive lung disease, bronchiectasis, alpha-1 antitrypsin deficiency, cystic fibrosis, granulomatous angiitis, rheumatoid arthritis, and gout[44–51]. Pharmacological inhibition of neutrophil serine proteases directly or via cathepsin C inhibition is currently explored in numerous pre-clinical and clinical trials and presents an attractive method of preventing tissue destruction in these diseases[44,49]. More so, since the neutrophil serine proteases do not seem to be essential for immune defense in humans[30] as discussed above. So far, studies have shown potential of inhibiting elastase in patients with ARDS, bronchiectasis, and patients undergoing cardiac surgery[45,49,52,53]. *In vitro* findings[9,10] have strongly suggested that proHNP processing is dependent on neutrophil serine proteases. If so, pharmacological inhibition of neutrophil serine proteases would lead to lack of HNP thus impairing innate immunity. Our study clearly demonstrates that even complete inactivation of neutrophil serine proteases does not lead to suppression of HNP levels *in vivo*. Future studies of the proHNP processing pathway must therefore examine other protease classes as well.
